# Supplementary material for: Transformers for molecular property prediction: domain adaptation efficiently improves performance
Source: J Cheminform. 2026 Jul 29;18:107. doi: 10.1186/s13321-026-01252-z (PMC13430829; doi:10.1186/s13321-026-01252-z)
Supplement: Supplementary file 1 — Supplementary material 1. [file 13321_2026_1252_MOESM1_ESM.pdf]

Appendix A Supporting Information

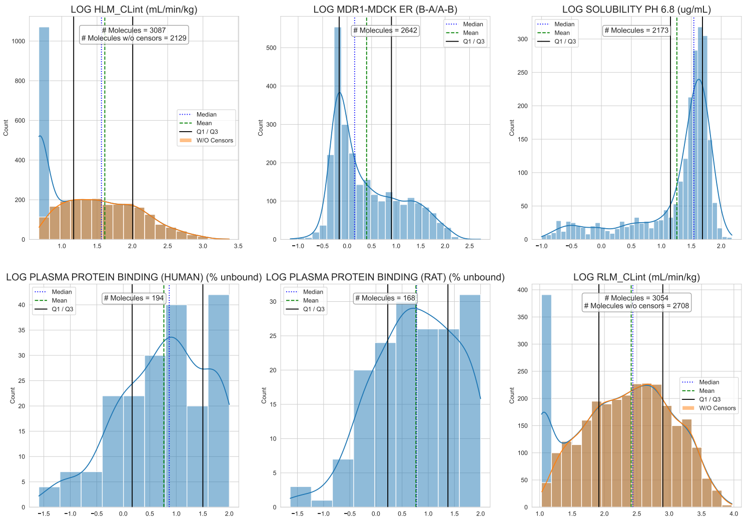

Fig. S1 Fang *et. al.* [3] dataset summary and distribution.

Dataset	# Molecules	# Unique scaffolds	% Unique scaffolds	**ADME ∩ GuacaMol (%)**	
				Common Mols	Common scaffolds
GuacaMol	1,273,104	407,408	32.00	–	–
Lipophilicity	4,196	2,440	58.15	56.79	81.02
$\text{Solubility}_{\text{AZ}}$	1,763	1,067	60.52	61.03	85.19
HPPB	1,614	1,044	64.68	50.06	79.31
Permeability	2,642	2,319	87.77	6.85	35.66
$\text{Solubility}_{\text{Fang}}$	2,173	1,885	86.75	7.96	39.68
HLM	3,087	2,658	86.10	7.19	36.08
RLM	3,054	2,626	85.99	7.17	36.25

Table S1 Downstream ADME dataset overlap statistics with the pre-training GuacaMol dataset, reporting molecule counts, unique Murcko scaffolds, and the percentage overlap (common molecules and scaffolds) between each ADME dataset and GuacaMol.

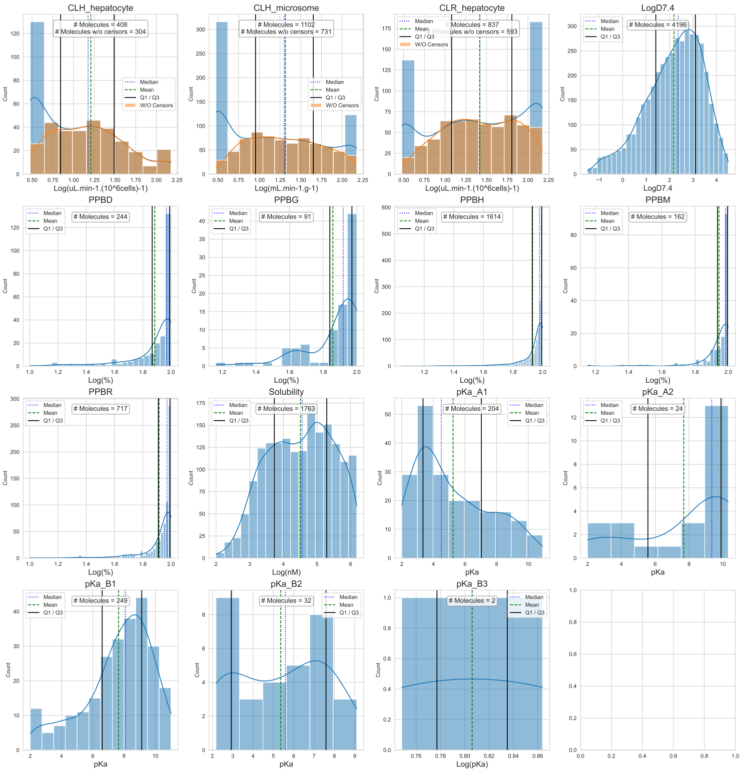

Fig. S2 AstraZeneca ChEMBL [31] dataset summary and distribution.

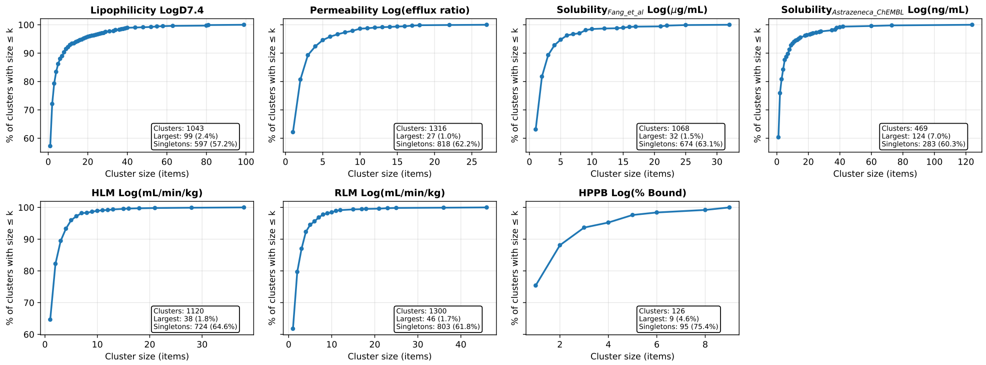

Fig. S3 Distribution of the sizes of the Butina clusters. For each of the downstream datasets we run clustering as detailed in section 3.4. We plot the fraction of clusters that have more than k members. We can observe that most of the clusters contain only a few molecules. The large number of small clusters can be explained by the diversity in the datasets, since a high similarity threshold was used for clustering (0.65).

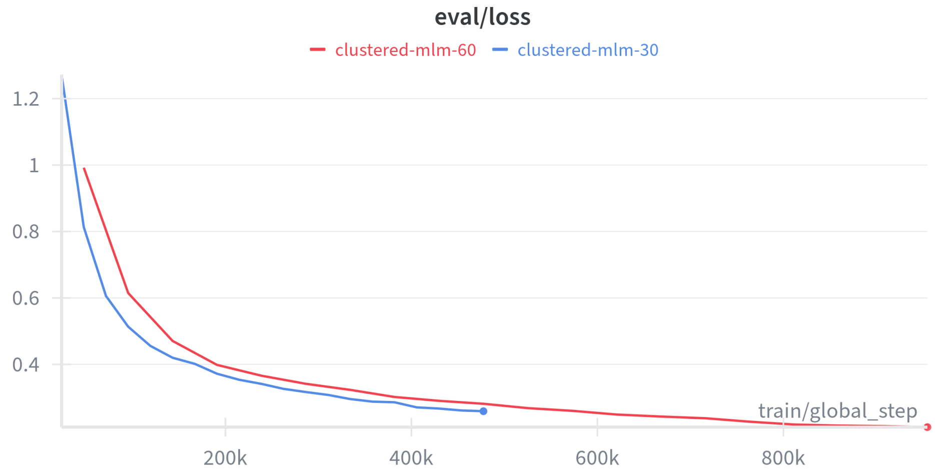

Fig. S4 MLM validation loss during pre-training. Although the models do not reach full convergence, we observed that further reducing the pre-training validation loss, e.g., decreasing it from 0.3 to 0.2, would require an exponentially increasing number of additional training steps. Given this behavior, and considering that domain adaptation (e.g., via MTR) yields substantial performance improvements, we argue that investing computational effort in domain adaptation is more beneficial than extending pre-training. This is especially true in light of the diminishing returns expected from additional optimization on the pre-training objective.

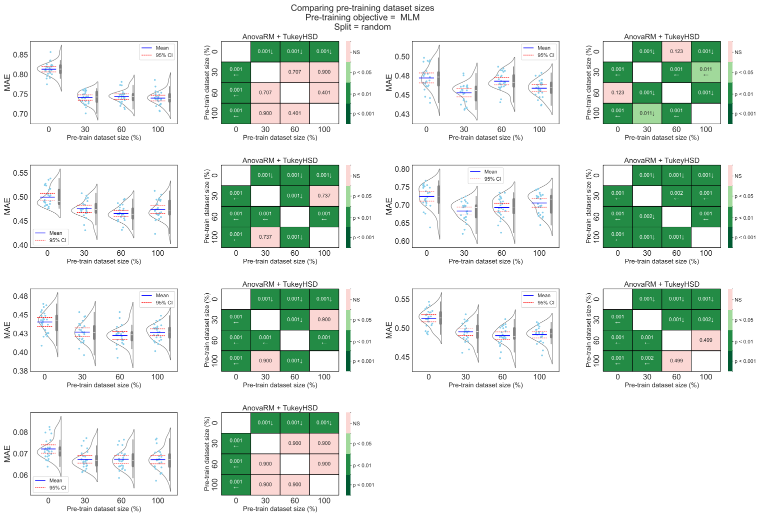

Fig. S5 Random splitting results for MAE performance for increasing pre-training dataset size. 0% corresponds to a randomly initialized model with no pre-training and 100% correspond to the ∼1.3M molecules of the GuacaMol dataset. Two-tailed significance analysis were performed, therefore, the arrows in the heatmap helps recognizing the model with the improved performance. CI = confidence interval for the estimation of the mean.

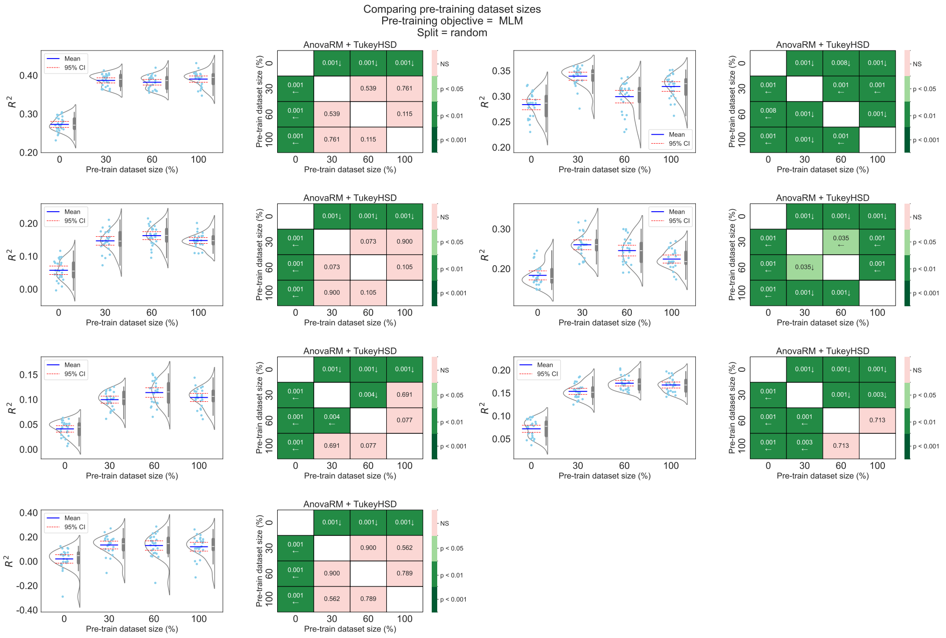

Fig. S6 Random splitting results for R^2 performance for increasing pre-training dataset size. 0% corresponds to a randomly initialized model with no pre-training and 100% correspond to the ∼1.3M molecules of the GuacaMol dataset. Two-tailed significance analysis were performed, therefore, the arrows in the heatmap helps recognizing the model with the improved performance. CI = confidence interval for the estimation of the mean.

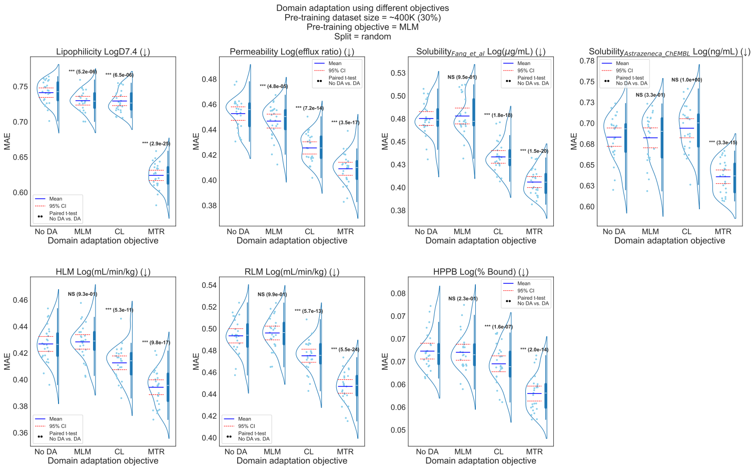

Fig. S7 Random splitting results for MAE performance for a baseline model trained with pre-training only (No DA), and three models incorporating domain adaptation (DA) using different objectives: Masked Language Modeling (MLM), Contrastive Learning (CL), and Multi-task Regression (MTR) for RDKit descriptors. P-values are from one-tailed paired t-tests comparing each DA model to the No DA baseline, under the hypothesis that DA improves performance. Significance levels: * $p < 0.05$, ** $p < 0.01$, *** $p < 0.001$.

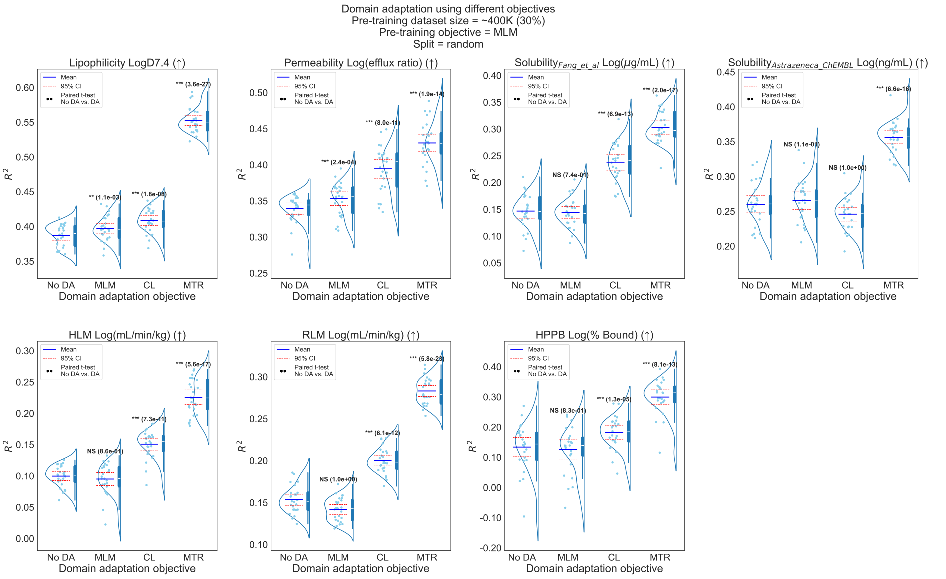

Fig. S8 Random splitting results for R^2 performance for a baseline model trained with pre-training only (No DA), and three models incorporating domain adaptation (DA) using different objectives: Masked Language Modeling (MLM), Contrastive Learning (CL), and Multi-task Regression (MTR) for RDKit descriptors. P-values are from one-tailed paired t-tests comparing each DA model to the No DA baseline, under the hypothesis that DA improves performance. Significance levels: * $p < 0.05$, ** $p < 0.01$, *** $p < 0.001$.

Category	#	Descriptors
Molecular Weight and Composition	8	MolWt, ExactMolWt, HeavyAtomMolWt, HeavyAtomCount, NumValenceElectrons, NumRadicalElectrons, FractionCSP3, NumHeteroatoms
Structural Count	17	RingCount, HeavyAtomCount, NHOHCount, NOCount, NumAliphaticCarbocycles, NumAliphaticHeterocycles, NumAliphaticRings, NumAromaticCarbocycles, NumAromaticHeterocycles, NumAromaticRings, NumHAcceptors, NumHDonors, NumHeteroatoms, NumRotatableBonds, NumSaturatedCarbocycles, NumSaturatedHeterocycles, NumSaturatedRings
Functional Groups Counts	85	fr_ prefix (e.g., fr_benzene, fr_alkyl_halide, fr_amide, etc.)
Drug likeness	1	QED
Electronic and Partial Charge	11	MaxAbsEStateIndex, MaxEStateIndex, MinAbsEStateIndex, MinEStateIndex, MaxPartialCharge, MinPartialCharge, MaxAbsPartialCharge, MinAbsPartialCharge, NumHAcceptors, NumHDonors, NumValenceElectrons
Lipophilicity and Refractivity	24	MolLogP, MolMR, SlogP_VSA1--SlogP_VSA12, SMR_VSA1--SMR_VSA10
Polar Surface Area	38	TPSA, LabuteASA, PEOE_VSA1--PEOE_VSA14, EState_VSA1--EState_VSA11, VSA_EState1--VSA_EState10
Topological Indices and Complexity	31	Chi0, Chi0n, Chi0v, Chi1, Chi1n, Chi1v, Chi2n, Chi2v, Chi3n, Chi3v, Chi4n, Chi4v, Kappa1, Kappa2, Kappa3, HallKierAlpha, BalabanJ, BertzCT, AvgIpc, BCUT2D_MWHI, BCUT2D_MWLOW, BCUT2D_CHGHI, BCUT2D_CHGLO, BCUT2D_LOGPHI, BCUT2D_LOGPLOW, BCUT2D_MRHI, BCUT2D_MRLOW, SPS, FpDensityMorgan1, FpDensityMorgan2, FpDensityMorgan3
**Total**	**209**	

Table S2 RDKit Descriptor categories and counts (total 209 descriptors). Some descriptors are repeated across categories because they belong to both of them. E.g., HeavyAtomCount appears in Molecular Weight and Composition and Structural Count. The total number counts such a feature only once. While the original list of RDKit contains 210 descriptors, we dropped the Ipc descriptor because it introduces numerical instabilities that cause model failures.

**Parameter**	**Default value**
bootstrap	True
ccp_alpha	0.0
criterion	squared_error
max_depth	None
max_features	1.0
max_leaf_nodes	None
max_samples	None
min_impurity_decrease	0.0
min_samples_leaf	1
min_samples_split	2
min_weight_fraction_leaf	0.0
n_estimators	100
n_jobs	None
oob_score	False
random_state	None
verbose	0
warm_start	False

Table S3 Default parameters for `sklearn.ensemble.RandomForestRegressor` `version 1.2.0`.

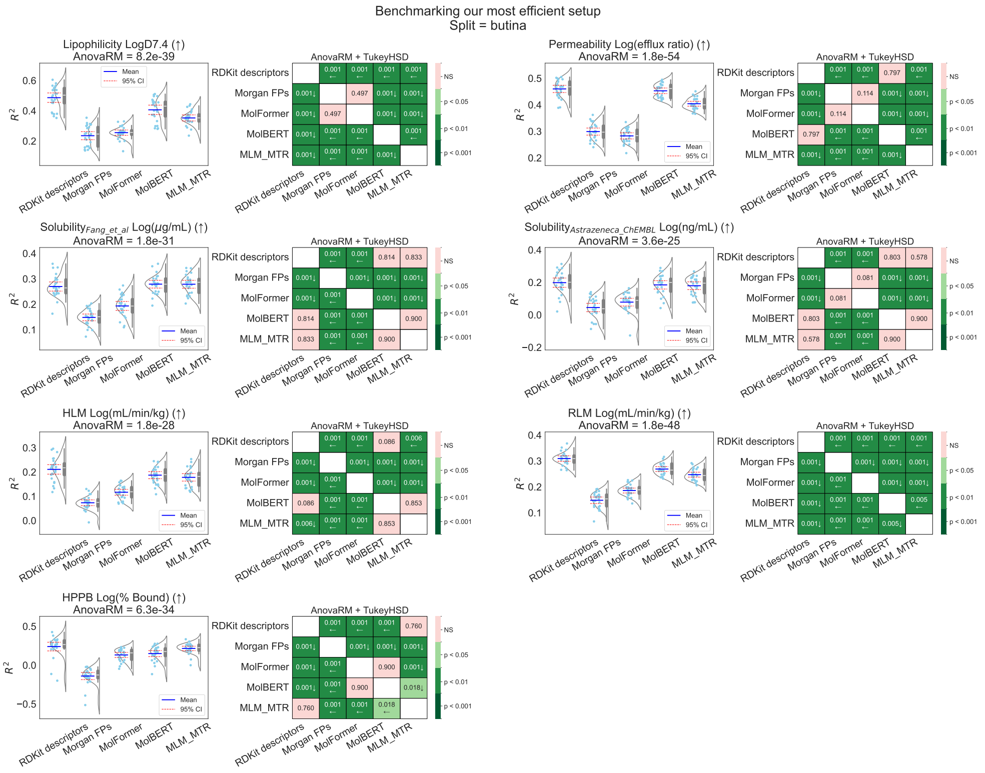

Fig. S9 R^2 performance of the most efficient model from our analysis to models from the literature. MLM_MTR corresponds to a transformer model pre-trained with $\sim$ 400K molecules using the MLM objective then domain adapted on the corresponding endpoint using the MTR objective. MolBERT [20] is a chemically aware transformer than has been pre-trained on $\sim$ 1.3M molecules using MLM, MTR, and SMILES-EQ objectives. MolFormer [26] is a large-scale transformer pre-trained on 100M molecules using MLM. RDKit descriptors and Morgan fingerprint correspond to two baseline representation. Random forest model was trained using all these representations. Two-tailed significance analysis were performed, therefore, the arrows in the heatmap helps recognizing the model with the improved performance. CI = confidence interval for the estimation of the mean.
